# Evaluation of the impact of nasal colonization with methicillin-resistant *Staphylococcus aureus* on left ventricular assist device infections

**DOI:** 10.1017/ash.2022.46

**Published:** 2022-05-10

**Authors:** Lauren J. Koscal, Holly Meadows, Brian R. Raux, Krutika N. Mediwala, Erin Weeda, Brian Houston, Deeksha Jandhyala, Jaclyn M. Hawn

**Affiliations:** 1 Department of Pharmacy, University of Florida Health at Jacksonville, Jacksonville, Florida; 2 Department of Pharmacy, Medical University of South Carolina, Charleston, South Carolina; 3 Medical University of South Carolina, College of Pharmacy, Charleston, South Carolina; 4 Division of Cardiology, Department of Medicine, Medical University of South Carolina, Charleston, South Carolina; 5 Division of Infectious Diseases, Department of Medicine, Medical University of South Carolina, Charleston, South Carolina

## Abstract

In this retrospective cohort study, we evaluated the predictive value of methicillin-resistant *Staphylococcus aureus* (MRSA) nasal swabs for the development of MRSA infections in patients with left ventricular assist devices. In 106 patients, the MRSA nasal swab had a negative predictive value of 92.9% demonstrating a potential role in antibiotic de-escalation.

Heart failure remains the leading cause of morbidity and mortality in the United States.^1^ The utilization of durable mechanical circulatory support (MCS) has provided a life-saving means for patients by providing hemodynamic support as a bridge to transplant, bridge to decision, or destination therapy.^2^ Over the past decade, ∼26,688 continuous-flow left ventricular assist devices (LVAD) have been implanted in the United States.^3^ Long-term complications of durable MCS devices include stroke, bleeding, and infection. Infections in these patients may ultimately lead to increased hospital admissions, morbidity, and mortality.^2,4–6^ Nasal colonization with methicillin-resistant *S. aureus* (MRSA) is a risk factor for development of clinical MRSA infections.^7,8^ Thus, 94% of LVAD centers perform nasal colonization surveillance swabs for MRSA and 92% use antiseptics to reduce colonizing bacteria before MCS implant surgery.^2^ Despite the frequent surveillance of MRSA nasal colonization, limited studies have been conducted assessing its effectiveness and utility in the LVAD population. We evaluated the impact of MRSA nasal colonization on infectious outcomes in patients with an LVAD.

## Methods

This single-center, retrospective, cohort study was performed at a large, academic medical center. Patients were screened for inclusion if they were ≥18 years of age with an LVAD implantation between August 1, 2014, to July 1, 2019. These patients were evaluated for study outcomes from time of LVAD implantation until July 1, 2020. Patients were excluded if they had a positive MRSA nasal swab within 7 days prior to LVAD implantation and received MRSA decolonization, if they died during index hospitalization, if they had a nondurable MCS, or if no MRSA nasal swab was obtained within 365 days of their LVAD implantation.

The primary outcome of this study was to determine the prognostic value of MRSA nasal swabs for the development of MRSA LVAD-specific or LVAD-related infections by evaluating sensitivity, specificity, positive predictive value (PPV), and negative predictive value (NPV). The secondary outcomes were to assess the prevalence of LVAD-specific or LVAD-related infections, time to infection, hospital readmission rate, and mortality rate. A comprehensive electronic medical record review was conducted and data collection was performed by manual chart review. Baseline characteristics were assessed during the index admission for LVAD implantation. Outcomes were assessed from the time of first positive MRSA nasal swab following LVAD implantation or first negative MRSA nasal swab if a positive was not detected.

## Definitions

An infection was defined according to the International Society for Heart and Lung Transplantation (ISHLT) criteria as the development of a positive bacterial culture at our institution.^2^ An LVAD-specific infection involved any aspect of the device, including pump, cannula, pocket, or driveline. An LVAD-related infection occurs in patients without MCS, including endocarditis, mediastinitis, bacteremia, and intra-abdominal infection.^2^ MRSA nasal swab surveillance and routine culture collection were not obtained at regular intervals at our institution. Additionally, methicillin-sensitive *S. aureus* (MSSA) nasal swab surveillance was not available during the study period. MRSA decolonization was defined as the use of mupirocin ointment or chlorhexidine solution prior to LVAD implantation, and no standardized protocol was implemented.^2^

### Statistical analysis

Statistical analyses were conducted using SPSS version 22 software (IBM, Armonk, NY). Baseline characteristics were analyzed using descriptive statistics. Outcomes with dichotomous variables were analyzed using the Fisher exact test, and continuous variables were analyzed using the Mann-Whitney *U* test. For all analyses, a 2-sided *P* value <.05 was considered significant.

## Results

In total, 125 patients were screened for enrollment, with 106 patients included in the analysis. Baseline characteristics assessed during the index admission for LVAD implantation are displayed in Table 1. Overall, 6.6% patients had a positive MRSA nasal swab for a median follow-up period of 1.94 years. The MRSA nasal swab test demonstrated the following diagnostic performance characteristics for detecting culture proven MRSA: sensitivity (22.2%), specificity (94.8%), PPV (28.6%), and NPV (92.9%). Comparing positive and negative MRSA nasal swabs, we detected similar rates of LVAD-related or LVAD-specific infections, and we detected no difference in additional secondary outcomes (Table 2). Moreover, 52 patients (49.1%) received MRSA decolonization prior to LVAD implantation, and among these patients, 5.6% had a subsequent positive MRSA nasal swab. We detected a higher prevalence of MRSA infection development in patients who did not receive MRSA decolonization (13.5%) compared to those who did (3.7%).

**Table 1.** Baseline Characteristics

Characteristic	Total (n= 106)
Age, median y (IQR)	53 (44–60)
Sex, male, no. (%)	70 (66.0)
Weight, median kg (IQR)	93 (77–108)
Height, median cm (IQR)	175 (168–183)
BMI, median kg/m^2^ (IQR)	29 (26–35)
**Ethnicity, no. (%)**	
African American	59 (55.7)
White	44 (41.5)
Other	3 (2.8)
Diabetes, no. (%)	48 (45.3)
WBC, median K/mm^3^ (IQR)	7.5 (5.9–9.8)
Serum creatinine, median mg/dL (IQR)	1.4 (1.1–1.9)
Renal replacement therapy, no. (%)	17 (16.0)
eGFR, median mL/min/1.73 m^2^ (IQR)	53 (32–72)
**CKD category, no. (%)**	
Stage 1	12 (11.3)
Stage 2	27 (25.5)
Stage 3a	18 (17.0)
Stage 3b	3 (2.8)
Stage 4	29 (27.4)
Stage 5	17 (16.0)
**Cause of heart failure, no. (%)**	
Ischemic cardiomyopathy	27 (25.5)
Nonischemic cardiomyopathy	74 (69.8)
Postpartum cardiomyopathy	5 (4.7)
**LVAD type, no. (%)**	
HeartMate II	46 (43.4)
HeartMate 3	41 (38.7)
HVAD	19 (17.9)
Destination therapy	86 (81.1)
Bridge to transplant	20 (18.9)
Time to extubation, median d (IQR)	0 (1–2)
MRSA decolonization, no. (%)	52 (49.1)
Any culture ordered, no. (%)	69 (65.1)
Blood culture ordered, no. (%)	59 (55.7)
**Positive blood culture (n = 59), no. (%)**	6 (10.2)
MRSA	0 (0)
MSSA	1 (16.7)
Other	5 (83.3)
Discharged on oral antibiotic, no. (%)	7 (6.6)
Discharged on intravenous antibiotic, no. (%)	2 (1.9)
Follow-up period, median y (IQR)	1.94 (1.05–3.07)

Note. BMI, body mass index; WBC, white blood cell count; eGFR, estimated glomerular filtration rate; CKD, chronic kidney disease; LVAD, left ventricular assist device; MRSA, methicillin-resistant *Staphylococcus aureus*; MSSA, methicillin-sensitive *Staphylococcus aureus*, IQR, interquartile range.

All baseline characteristics were obtained from index hospitalization for LVAD implantation.

**Table 2.** Secondary Outcomes

Outcome	Positive MRSA swab (n = 7)	Negative MRSA swab (n = 99)	*P* Value
LVAD-related or LVAD-specific infection, no. (%)	4 (57.1)	57 (57.6)	>.99
Time to infection, median d (IQR)	37 (5–205)	226 (121–437)	.122
**Infection type, no. (%)^a^**			
Driveline	2 (50.0)	36 (63.2)	.628
Pocket	0 (0)	1 (1.8)	>.99
Blood	1 (25.0)	11 (19.3)	>.99
Other	1 (25.0)	9 (15.8)	.521
MRSA infection, no. (%)	2 (28.6)	7 (7.1)	.108
MSSA infection, no. (%)	0 (0)	24 (24.2)	.346
Surgical intervention, no. (%)^a^	0 (0)	4 (7.0)	>.99
Discharged on oral antibiotics, no. (%)^a^	1 (25.0)	33 (57.9)	.313
Discharged on intravenous antibiotics, no. (%)^a^	2 (50.0)	17 (29.8)	.582
Subsequent infection-related hospital readmissions median (IQR)	1 (0–4)	0 (0–2)	.209
Hospital readmissions, median (IQR)	6 (3–8)	4 (2–8)	.333
Heart transplant, no. (%)	1 (14.3)	23 (23.2)	>.99
Mortality, no. (%)	3 (42.9)	26 (26.3)	.389
Lost to follow up, no. (%)	0 (0)	3 (3.0)	>.99

Note. MRSA, methicillin-resistant *Staphylococcus aureus*; LVAD, left ventricular assist device; MSSA, methicillin-sensitive *Staphylococcus aureus*; IQR, interquartile range

a In the analysis, 4 patients had a positive MRSA swab and 57 patients had a negative MRSA swab.

## Discussion

This study was designed to determine the utility of MRSA nasal colonization as a risk factor for the development of LVAD-related or LVAD-specific infections. The use of MRSA nasal screening to rule out MRSA respiratory infections has led to improvements in early antimicrobial de-escalation. However, its clinical utility in other infection types remains unclear. Thus far, no studies assessed the predictive value of MRSA infections in patients with LVADs.^7,8^ In 82 patients who received an LVAD, Papathanasiou et al^9^ found that colonization with a multidrug-resistant bacteria prior to implantation was associated with higher likelihood of death from infection in 34.1% of patients. However, only 7 of these patients were colonized with MRSA.^9^ Nurjadi et al^10^ evaluated nasal colonization with *S. aureus* in 49 patients with durable ventricular assist devices and found that *S. aureus* nasal colonization was associated with an increased incidence of infection by *S. aureus* during the first year after implantation. In comparison to these studies, our study focused on determining the clinical utility of MRSA nasal swabs for predicting specifically MRSA infections. We included a larger sample size, and our study had a longer follow-up period. Although the MRSA nasal swab demonstrated low sensitivity, its high NPV supports the use of MRSA nasal swabs as a tool for early antimicrobial de-escalation in a patient population at high risk for developing drug resistance. In our study, 7 patients with a negative MRSA nasal swab went on to develop a MRSA infection; however, the average time from collection to MRSA infection was 178 days. This finding highlights the importance of implementing standardized surveillance.

The 2015 ISHLT guidelines for prevention and management strategies for MCS infection recommend considering utilization of mupirocin and chlorhexidine before MCS implantation for patients with MRSA colonization.^2^ Our study showed that patients who received MRSA decolonization had a lower prevalence of development of a MRSA infection. MRSA decolonization was implemented with the use of either mupirocin ointment or chlorhexidine solution, which are relatively inexpensive interventions, suggesting a benefit of implementing universal decolonization protocols.

This study had several limitations. Due to the retrospective study design, our results were limited by its observational nature. Microbiologic data were also limited to our institution, allowing for the possibility of unidentified infection development and outcomes. MRSA nasal swab surveillance was not consistent during the study period, and MSSA nasal swab surveillance was not available. Future studies should be conducted to address these limitations, including implementation of MSSA nasal swab surveillance, routine nasal swab screenings, and standardization of decolonization.

For patients with an LVAD, our study demonstrated that MRSA nasal swabs have a high NPV for detecting LVAD-specific and LVAD-related MRSA infections. Overall, the utilization of MRSA nasal swabs for de-escalation of antimicrobial therapy for LVAD-associated infections should be considered. Additionally, universal MRSA decolonization may have a role in improving outcomes in this patient population. More studies are needed to definitively assess the utilization of MRSA nasal swabs in patients with LVADs.
